# Contribution of clinical trials to gross domestic product in Hungary

**DOI:** 10.3325/cmj.2014.55.446

**Published:** 2014-10

**Authors:** Zoltán Kaló, János Antal, Miklós Pénzes, Csilla Pozsgay, Zsuzsanna Szepezdi, László Nagyjánosi

**Affiliations:** 1 Eötvös Loránd University (ELTE), Department of Health Policy and Health Economics, Budapest, Hungary; 2 Parexel Hungary Ltd, Budapest, Hungary; 3 MSD Pharma Hungary, Budapest, Hungary; 4 Egis Pharmaceuticals Plc., Budapest, Hungary; 5 Syreon Research Institute, Budapest, Hungary

## Abstract

**Aim:**

To determine the contribution of clinical trials to the gross domestic product (GDP) in Hungary.

**Methods:**

An anonymous survey of pharmaceutical companies and clinical research organizations (CROs) was conducted to estimate their clinical trial-related employment and revenues. Clinical trial documents at the National Institute of Pharmacy (NIP) were analyzed to estimate trial-related revenues at health care institutions and the value of investigational medical products (IMPs) based on avoided drug costs. Financial benefits were calculated as 2010 US $ purchasing power parity (PPP) values.

**Results:**

Clinical trials increased the revenue of Hungarian health care providers by US $165.6 million. The value of IMPs was US $67.0 million. Clinical trial operation and management activities generated 900 jobs and US $166.9 million in revenue among CROs and pharmaceutical companies.

**Conclusions:**

The contribution of clinical trials to the Hungarian GDP in 2010 amounted to 0.2%. Participation in international clinical trials may result in health, financial, and intangible benefits that contribute to the sustainability of health care systems, especially in countries with severe resource constraints. Although a conservative approach was employed to estimate the economic benefits of clinical trials, further research is necessary to improve the generalizability of our findings.

Active participation in international clinical trials may provide health benefits to patients and financial and professional benefits to health care providers. In lower income economies, such as those in Central-Eastern Europe (CEE), the relative benefits of clinical trials are even greater than in the high income countries of Western Europe and North America. Consequently, the contribution of emerging markets to international clinical trials is growing substantially (1). This phenomenon is especially visible in CEE, where the number of clinical trials has increased significantly over the past 15 years and is expected to increase even further in the near future (2). In CEE, international clinical trials offer opportunities for site personnel to improve their professional networking and be remunerated on higher-than average income level. For health care institutions with substantial budget constraints, trial-related payments can represent an important source of liquid cash. A supportive attitude of hospital management toward clinical trial activities, in terms of providing better working environment or increased remuneration, may help to prevent the migration of qualified professional staff to higher income countries. In CEE countries, the health status of the population is worse than in higher income Western European countries (3) and the accessibility of new medicines is relatively limited (4). Therefore, through clinical trials, CEE patients can obtain access to standardized modern health care services, technologies, and investigational drugs without waiting lists or co-payments. However, investigational medical products (IMPs) may represent considerable health risks for patients.

The societal gain associated with clinical trials is multifactorial. Clinical trials contribute to the evolution of evidence-based medicine. They systematically investigate side effects and health outcomes not only for IMPs but also for the control treatment arms. Therefore, safety information, even about marketed therapies, is captured and no public investment is necessary.

The most tangible benefit may be the financial impact, including the contribution of trials to the revenues of health care providers and clinical research organizations (CROs). However, there are also indirect benefits, such as avoided health care expenses due to the free delivery of IMPs and services.

Few scientific publications have addressed the financial benefits of clinical trials. These publications examined avoided drug costs and additional revenues primarily from the viewpoint of health care institutions (5-9). There is also one Polish study on the national economic impact of clinical trials, but the approach was not comprehensive enough to capture all direct and indirect financial benefits (10).

Hungary currently has a favorable position for implementation of clinical trials (11). It has high-level professionalism at investigational centers, rapid regulatory and ethical endorsements of applications, complex but manageable contracting processes at clinical sites, sufficient contributions to patient recruitment, and high Good Clinical Practice (GCP) quality according to Food and Drug Administration (FDA) inspections (12). However, similarly to other CEE countries, the capacity for clinical trial participation in Hungary has not been maximized. The aim of this study was to determine the contribution of clinical trials to the national economy in Hungary. We estimated the clinical trial-related revenues of CROs, investigators, and health care institutions and the financial benefits of avoided drug costs due to IMPs as the percentage of the gross domestic product (GDP).

## Methods

The economic impact of clinical trials was measured from several different perspectives. In 2009, the Hungarian Clinical Trial Management Society (CTMS) and the International Society for Pharmacoeconomics and Outcomes Research Hungary Chapter (ISPOR HCh) (13) obtained information about clinical trial-related revenues among health care institutions and CROs. In 2012, the ISPOR HCh collected additional information about the value of investigational drugs in clinical trials.

In the first step, to estimate the operational costs and the number of clinical research associates and other medical professionals involved in clinical trial activities in 2008, the CTMS conducted an anonymous survey among CRO managers with operations in Hungary and medical directors at research-based pharmaceutical companies. The questionnaire was mailed three times to 65 companies, and 12 questionnaires with a full set of data were returned. The aggregate survey results were assumed to be proportional to the total.

Information on clinical trial-related revenues and employment was validated and consolidated based on the annual balance sheets of Hungarian CROs for the 2008 fiscal year. In Hungary, public and private companies are obliged to provide annual financial data, and the Court of Registration makes these reports publicly available.

In the second step, after having signed the National Institute of Pharmacy (NIP) confidentiality agreement, two health economists from the ISPOR HCh reviewed the master files of clinical trials that were approved in 2008. The NIP approves and controls clinical trials in Hungary and archives master files of all interventional clinical trials, including information on trial budget estimates. The researchers assessed a randomly selected sample of clinical trial master files that were submitted to the NIP for approval. They calculated the total clinical site-related budgets of the clinical trials, including investigator fees and institutional costs. In total, 313 clinical trial applications were approved. Of 59 randomly selected studies, 9 files were excluded because of insufficient information on the site-related budget. The 50 remaining trials were representative of the overall allocation of studies in different clinical trial phases (χ^2^ test *P* = 0.6) (Table 1).

**Table 1:** Development phases of clinical trials in the National Institute of Pharmacy appraisals (2008)

	Total approved trials in Hungary	Assessed clinical trials	Proportion of assessed trials relative to the total (%)
Phase I and bioequivalence	38	6	15.8
Phase II	94	11	11.7
Phase III	153	29	19.0
Phase IV	28	4	14.3
Total	313	50	16.0

As no information was available on the allocation of trial budgets by calendar year, we assumed that the clinical trial revenues of health care providers before a given calendar year were equal to their estimated revenues in subsequent years. This assumption was supported by the fact that the number of clinical trials in Hungary remained relatively constant from 2006-2011 (11).

Financial figures from 2008 were converted to 2010 Hungarian Forint (HUF) values using the consumer price indices for 2009 and 2010 (4.2% and 4.9%, respectively). The results in HUF were converted into US $ using the GDP-specific purchasing power parity (PPP) exchange rate in 2010 (US $1 = HUF 130.12).

The third step was to estimate the indirect value of IMPs based on avoided drug costs for patients treated in clinical trials. All phase II-IV trials that were licensed in Hungary in 2010 were selected. Phase I and bioequivalence studies were excluded because the participants are healthy volunteers without a need for treatment. After signing the confidentiality disclosure agreement, a health economist retrieved information from the clinical trial master files at the NIP from the clinical studies approved in 2010, including the European Clinical Trials database (EudraCT) number, detailed characteristics of the investigational compound and its comparator, the dosages of IMPs that were provided free of charge to study participants, and the full study protocol. From the EudraCT database, the following additional information was retrieved by the NIP experts (only authorized individuals are allowed to retrieve data from this international database): clinical trial authorization date, planned number of study participants in Hungary, and Medical Dictionary for Regulatory Activities (MedDRA) categories for the therapeutic area. The value of the investigational compounds was conservatively estimated based on the public price of the study comparator drug or a similar marketed product in the same ATC group or therapeutic area. The price was obtained from the drug list of the Hungarian National Health Insurance Fund (NHIF). In the three cases in which the Hungarian price for a first-in-class IMP or comparator was not available, German drug prices listed on *www.medizinfuchs.de* were used. The value of rescue medications was assumed to be zero because their use depends on IMPs and not on routine medical care. No additional technological costs were included in the value of IMPs (eg, additional diagnostics). Because the number of clinical trials and the proportions of different trial phases in Hungary had been constant in the years before the study, we assumed that the value of IMPs from clinical trials approved before a given calendar year was equal to the estimated value of IMPs in the subsequent years. In 2010, 262 phase II-IV clinical trial applications were approved. Fourteen clinical trials were excluded due to incomplete data. Therefore, the value of IMPs was estimated based on an analysis of 248 clinical trial master files.

The clinical trial master files contained information only on the planned number of patients. Actual recruitment is usually lower than the planned number due to competitive recruitment among countries. As the actual number of recruited patients was not known, 6 senior managers at different CROs were interviewed. Based on their consensus statement, the ratio of planned to actual recruitment was assumed to be 80%. Furthermore, the assumption was that 15% of the screened patients would drop out before treatment allocation and an additional 15% would drop out at the mid-point of the study.

The financial benefits of the clinical trials were calculated as HUF 2010 values and converted into US $ 2010 PPP values. Finally, the total financial value of the clinical trial-related benefits was compared with the Hungarian GDP in 2010.

## Results

Based on the CTMS survey and data from the annual balance sheets of CROs, approximately 900 professionals, or 1 out of every 4350 Hungarian employees, worked in clinical trial-related functions at CROs and pharmaceutical companies. Based on data from the CTMS survey, the total value of trial management activities was 166.9 million in 2010 US $. This amount included the gross income of clinical trial professionals at pharmaceutical companies and CROs and other operational costs, such as traveling, office and storage costs, communication and IT costs, and legal and financial counseling expenses, but excluded spending at clinical sites.

According to data from the NIP, the annual revenue of health care professionals and their institutions at clinical sites was 165.6 million in 2010 US $ (Table 2), which represented an additional 2.84% of revenues to NHIF-funded traditional health care services. A major proportion of clinical site-related revenues represented a personal income source for physicians and nurses.

**Table 2:** Site-related budgets of clinical trials in Hungary according to the National Institute of Pharmacy documentation

	Total budget of approved clinical trials in 2008 (millions, 2010 National Institute of Pharmacy purchasing power parity)
Phase I and bioequivalence	4.9
Phase II	39.7
Phase III	114.2
Phase IV	6.8
Total	165.6

Significant savings were generated from avoided drug costs for clinical trial participants. The estimated annual financial value of IMPs in phase II-IV clinical trials was US $67.0 million, which was equal to 2.52% of the NHIF pharmaceutical budget. Phase III trials accounted to 65% of the total amount, whereas phase II and phase IV trials accounted for 15% and 20%, respectively. Three disease areas (neoplasms, diseases of the nervous system, and musculoskeletal diseases) represented 75% of the total value of IMPs, although they included only 30% of enrolled patients.

The revenues of health care providers (ie, investigators, hospitals) and the clinical trial industry, and the value of IMPs together amounted to US $399.5 million, which was equal to 0.2% of the GDP (Table 3).

**Table 3:** Contribution of clinical trials to the Hungarian economy

	Monetary value (millions, 2010 National Institute of Pharmacy purchasing power parity)	Gross domestic product percentage
Revenues of clinical research organizations and pharmaceutical companies (excluding expenditures at research sites)	166.9	0.082
Clinical trial revenue of health care institutions and investigators	165.6	0.081
Value of investigational medical products	67.0	0.033
Total contribution of clinical trials to the economy	399.5	0.195

## Discussion

Hungarian physicians and patients have been participating in international clinical trials for more than twenty years. However, the related economic benefits have never been assessed. The shared effort of two professional associations, namely, the CTMS (clinical trial managers) and ISPOR HCh (health economics researchers), aims to provide real-world economic data to government officials and politicians in order to obtain strategic support for and engagement with the implementation of international clinical studies.

The three-step survey about the financial impact of clinical trials was based on the best available data, but the generalizability of our findings is limited due to several reasons. The sample size of the CTMS survey was relatively small. The review of annual balance sheets included CROs but excluded pharmaceutical companies, as clinical trial-related functions could not be separated from sales and marketing activities. The Directorate General of the NIP does not collect information on the number of patients who complete trials, so the 80% ratio of planned to actual patient recruitment and the average drop-out rate were based on expert opinions. However, Tufts Center also found a similar value of actual trial enrolment at Eastern European sites in 153 international phase II-III clinical trials (76%) (14). Furthermore, we could not capture the financial benefit related to improved health outcomes or the potential risks related to IMPs, or economic multiplicator effect of clinical trial activities. We also did not capture the revenues or costs related to capacity building of clinical research, including the implementation of GCP training for clinical site personnel and infrastructural development at research sites (eg, phase I research centers). In general, we employed a conservative approach to estimate the economic benefits of clinical trials.

In conclusion, participation in international clinical trials may result in health, financial, and intangible benefits that contribute to the sustainability of health care systems with limited resources. The direct financial benefit of clinical trials, in the form of the revenues of CROs and investigators, contributed to the Hungarian GDP by 0.163%, and avoiding pharmaceutical spending represented additional indirect benefits that accounted for 0.033% of the GDP. It is difficult to compare our findings to those from other countries as we are unaware of similar studies that have considered various aspects of clinical trial-related economic benefits.

Individual countries can strengthen their market position if policymakers, relevant authorities, and management teams at investigational sites support the implementation of clinical trials (2,15). Long-term national strategies in different areas, including postgraduate education, streamlined ethical and regulatory approval of trials, infrastructural development at trial sites, and the promotion of specific trial management skills and capacities, may improve the competitiveness of countries with severe health care resources constraints. Additional efforts should be made to develop regional cooperation in CEE. Countries with similar backgrounds and geographical and economic statuses can learn from the successful strategies of other countries and eventually improve the competitiveness of the entire CEE region in attracting clinical trials.
